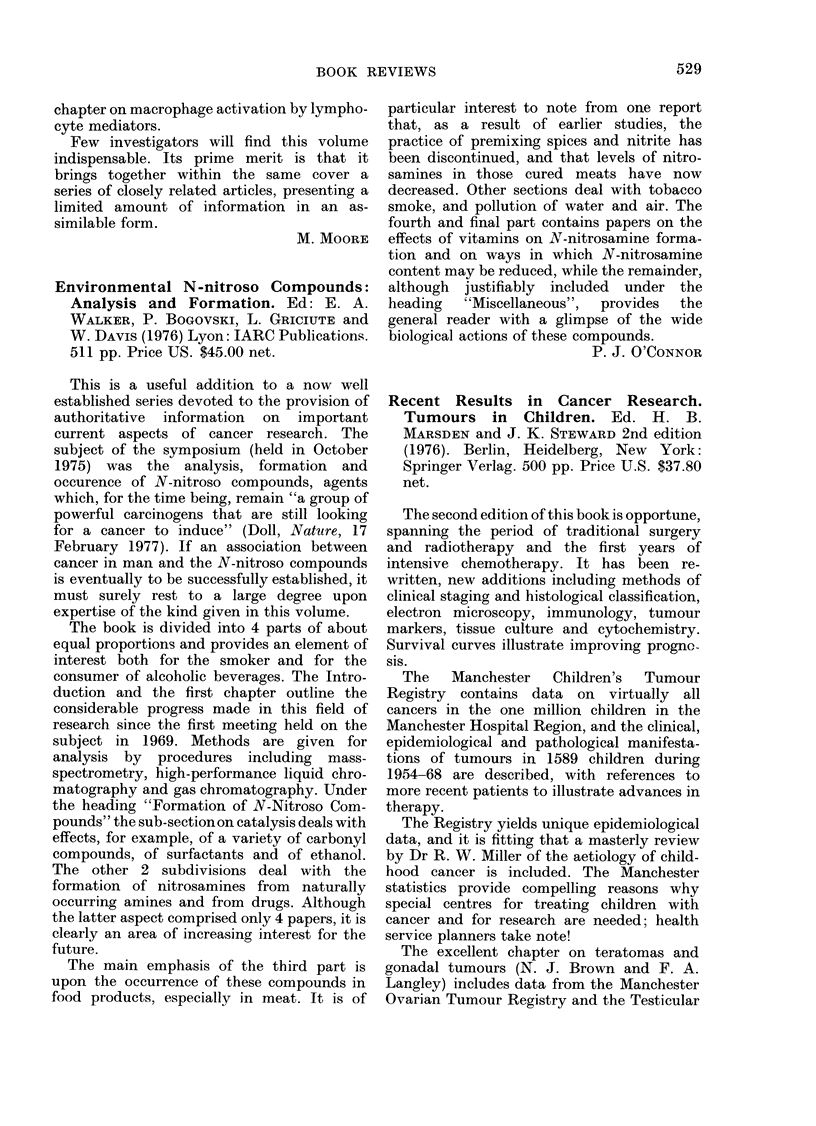# Environmental N-nitroso Compounds: Analysis and Formation

**Published:** 1977-10

**Authors:** P. J. O'Connor


**Environmental N-nitroso Compounds: Analysis and Formation.** Ed: E. A. WALKER, P. BOGOVSKI, L. GRICIUTE and W. DAVIS (1976) Lyon: IARC Publications. 511 pp. Price US. $45.00 net.

This is a useful addition to a now well established series devoted to the provision of authoritative information on important current aspects of cancer research. The subject of the symposium (held in October 1975) was the analysis, formation and occurence of *N*-nitroso compounds, agents which, for the time being, remain "a group of powerful carcinogens that are still looking for a cancer to induce" (Doll, *Nature*, 17 February 1977). If an association between cancer in man and the *N*-nitroso compounds is eventually to be successfully established, it must surely rest to a large degree upon expertise of the kind given in this volume.

The book is divided into 4 parts of about equal proportions and provides an element of interest both for the smoker and for the consumer of alcoholic beverages. The Introduction and the first chapter outline the considerable progress made in this field of research since the first meeting held on the subject in 1969. Methods are given for analysis by procedures including mass-spectrometry, high-performance liquid chromatography and gas chromatography. Under the heading "Formation of *N*-Nitroso Compounds" the sub-section on catalysis deals with effects, for example, of a variety of carbonyl compounds, of surfactants and of ethanol. The other 2 subdivisions deal with the formation of nitrosamines from naturally occurring amines and from drugs. Although the latter aspect comprised only 4 papers, it is clearly an area of increasing interest for the future.

The main emphasis of the third part is upon the occurrence of these compounds in food products, especially in meat. It is of particular interest to note from one report that, as a result of earlier studies, the practice of premixing spices and nitrite has been discontinued, and that levels of nitrosamines in those cured meats have now decreased. Other sections deal with tobacco smoke, and pollution of water and air. The fourth and final part contains papers on the effects of vitamins on *N*-nitrosamine formation and on ways in which *N*-nitrosamine content may be reduced, while the remainder, although justifiably included under the heading "Miscellaneous", provides the general reader with a glimpse of the wide biological actions of these compounds.

P. J. O'CONNOR